# Correlation of malaria parasitaemia with peripheral blood monocyte to lymphocyte ratio as indicator of susceptibility to severe malaria in Ghanaian children

**DOI:** 10.1186/s12936-018-2569-x

**Published:** 2018-11-12

**Authors:** Samuel Antwi-Baffour, Ransford Kyeremeh, Dorcas Buabeng, Jonathan Kofi Adjei, Claudia Aryeh, George Kpentey, Mahmood Abdulai Seidu

**Affiliations:** 1 Department of Medical Laboratory Sciences, School of Biomedical and Allied Health Sciences, College of Health Sciences, University of Ghana, P. O. Box KB 143, Korle-Bu, Accra, Ghana; 2 Central Laboratories, Korle-bu Teaching Hospital, Korle-bu, Accra, Ghana; 3 Department of Haematology, Sunyani Regional Hospital, Sunyani, Ghana; 4 Department of Haematology, Wa Regional Hospital, Wa, Ghana

**Keywords:** Monocytes, Lymphocytes, Parasitaemia, Malaria and peripheral blood

## Abstract

**Background:**

Even though malaria is generally on the decline due extensive control and elimination efforts, it still remains a public health problem for over 40% of the world’s population. During the course of malaria infection, parasites and red blood cells come under oxidative stress and there is host immune response in an attempt to protect the red blood cells. The frequency of monocytes and lymphocytes in peripheral blood might, therefore, be expected to reflect the state of an individual’s immune response to the infection. Circulating monocytes and lymphocytes could therefore serve as an index in relation to malaria parasitaemia. The purpose of this study was to determine whether the relative count of monocytes to lymphocytes in peripheral blood (M:L ratio) can predict parasitaemia and, therefore, the severity of malaria infection.

**Methods:**

Two millilitre of venous blood sample were taken from participants by venisection into anticoagulant tubes. Thick and thin blood films were made and stained with Giemsa and examined for malaria parasites. Whole blood specimen were analysed for full blood count using ABX Pentra 60 C+ automated haematological analyzer. Data was entered into Microsoft Word and analysed using Statistical Package for Social Sciences (SPSS, Version 20.0) and Graphpad prism. Spearman’s correlation was used to determine correlation between occurrences of clinical malaria and the monocytes and lymphocytes ratio. Statistical significance was taken as p ≤ 0.05 with 95% confidence interval.

**Results:**

The study comprised of 1629 (m = 896; f = 733) children up to 5 years presenting with clinical malaria as cases and 445 (m = 257; f = 188) apparently healthy children as controls. The results indicated that there was a significant positive correlation between the monocytes to lymphocytes ratio and the presence of parasites (p = 0.04) and the level of parasitaemia within the age group of 0–3 years (p = 0.02) and 4–5 years (p = 0.03).

**Conclusions:**

The monocyte to lymphocyte ratio obtained correlated positively with the presence of malaria as well as the level of parasitaemia. The outcome of this work implies that monocyte to lymphocyte ratio can be used to predict the level of parasitaemia and together with other factors, the development of severe malaria.

## Background

In spite of a declining trend in the number of cases and deaths over the last few years, malaria still causes significant mortality and morbidity and the bulk of the burden is observed in children under 5 years of age [1]. The severity of the disease in this age group is evident with malaria attacks leading to 1 million of cerebral malaria and 4 million cases of severe anaemia each year [2]. In fact, according to the World Health Organization (WHO), about 285,000 children died before their fifth birthdays in 2016 in Africa [1]. Malaria therefore remains the single largest cause of death in children in Africa [3]. The disease kills children in three ways; infection in pregnancy (low birth weight and preterm delivery), acute febrile illness (cerebral malaria, respiratory distress and hypoglycaemia) and chronic respiratory infection (severe anaemia) [4].

Four *Plasmodium* species have been known to cause malaria infection in humans namely; *Plasmodium falciparum*, *Plasmodium vivax*, *Plasmodium ovale* and *Plasmodium malariae*. However, more recently, *Plasmodium knowlesi*, which use to infect long-tailed and pig-tailed macaque monkeys, has been implicated as a cause of human malaria in Southeast Asia [5]. Among the species, *P. falciparum* is the most common in sub-Saharan Africa and also responsible for the majority of severe malaria and malaria deaths globally. Severe malaria is defined as acute malaria with signs of organ dysfunction and/or high level of parasitaemia [6]. It is also defined by the demonstration of asexual forms of the malaria parasites in the blood in a patient with a potentially fatal manifestation or complication of malaria in whom other diagnoses have been excluded [7, 8]. The presentation of severe malaria however varies with age and geographical distribution and so in areas of high malaria transmission, severe malaria mainly affects children under 5 years of age [8, 9]. The remaining species are not typically as life-threatening as *P. falciparum* [10]. *Plasmodium vivax* is the second most significant specie and is prevalent in Southeast Asia and Latin America. Furthermore, *P. vivax* and *P. ovale* have an added dormant liver stage in their life cycle, which can be reactivated in the absence of a mosquito bite, leading to clinical symptoms [11].

Haematological changes are most common in malaria infections and these changes play a major role in the malaria pathology. These changes affect the major cell lines such as red blood cells, leukocytes and thrombocytes [12]. In a study conducted by Halim et al. [13] there was marked increase in monocytes count in individuals with malaria infections as monocytes tend to become phagocytic in the presence of protozoa, bacteria or fungi infections. Malaria infections also induce lymphocytopenia which is accompanied by an increase in neutrophil count which is usually considered as a sign of systemic inflammation [14].

Similarly, in Kenya, Warimwe et al. [15] showed that children with asymptomatic *P. falciparum* infection recorded high monocyte to lymphocyte ratio in clinical malaria during follow-up. This is because following repeated natural exposure to malaria infection people may not develop symptomatic malaria but will harbor the parasite which will continuously stimulate the immune system leading to the increase in monocyte numbers. Older children and adults therefore remain susceptible to asymptomatic *Plasmodium* infections, often caused by *P. falciparum* to which immunity probably never occurs [15].

The 2015, guidelines for the treatment of malaria and 2018 overview of malaria treatment by the WHO recommends presumptive diagnosis and treatment of children under age of five in endemic areas, only when microscopy or malaria rapid diagnostic test are unavailable [16]. However, this procedure can be supported by haematological analyses and parameters when available [17]. During the course of malaria infection, parasites and red blood cells come under oxidative stress and there is host immune response in terms of changes in monocyte and lymphocyte population in attempt to protect the red blood cells. The frequency of monocytes and lymphocytes in peripheral blood might therefore be expected to reflect the state of an individual’s immune response to infections [18]. This opens up the possibility that circulating leukocytes particularly lymphocytes and monocytes could serve as an index in relation to malaria parasitaemia and subsequently the development of severe malaria [19]. It is therefore essential to determine the monocyte to lymphocyte ratio during malaria infection, particularly in children under five as a measure of the level of parasitaemia and or severity of malaria.

It is a known fact that full blood and differential cell counts from the circulatory blood have been used routinely to aid in clinical diagnosis of a wide range of infectious diseases such as pneumonia and human immunodeficiency virus (HIV) as well as malaria in several studies [20–22]. They have also been used as a correlate of disease risk in some longitudinal studies [15]. In this work, full differential blood count results from Ghanaian children up to 5 years across three regional hospitals, was used to determine whether the relative count of monocytes to lymphocytes in peripheral blood (M:L ratio) can predict parasitaemia and together with other factors, the severity of malaria infection.

## Methods

### Study design

The study was a case control study conducted between the years 2014 and 2017.

### Setting of the study

The study was conducted across Ghana in three regional hospitals namely: the Korle-bu Teaching Hospital for the southern sector, the Sunyani Regional Hospital for the middle sector and the Wa Regional Hospital to cover the northern sector of the country.

### Sample size calculation

The sample sizes for the different study sites were calculated using the following formula:

$${\text{N}} = {\text{z}}^{2} \;{\text{p}}\;{{\left( {1 - {\text{p}}} \right)} \mathord{\left/ {\vphantom {{\left( {1 - {\text{p}}} \right)} {\text{e}}}} \right. \kern-0pt} {\text{e}}}^{2}$$

where z is the confidence interval which is set at 95% (z-value of 1.96); p is the average annual percentage prevalence of malaria cases among children which is 21% for Sunyani municipality, 16% for Accra municipality for Korle-bu Teaching Hospital and 10% for Wa municipality (from available regional statistics). e is the allowable error margin which is set 3%.

Calculation:

$${\text{For Sunyani, n}} = 1.96^{2} \times 0.21 \, {{\left( {1 - 0.21} \right)} \mathord{\left/ {\vphantom {{\left( {1 - 0.21} \right)} {0.03^{2} }}} \right. \kern-0pt} {0.03^{2} }} = 708$$

$${\text{For KBTH}},{\text{ n}} = 1.96^{2} \times 0.16 \, {{\left( {1 - 0.16} \right)} \mathord{\left/ {\vphantom {{\left( {1 - 0.16} \right)} {0.03^{2} }}} \right. \kern-0pt} {0.03^{2} }} = 574$$

$${\text{For Wa}},{\text{ n}} = 1.96^{2} \times 0.10 \, {{\left( {1 - 0.10} \right)} \mathord{\left/ {\vphantom {{\left( {1 - 0.10} \right)} {0.03^{2} }}} \right. \kern-0pt} {0.03^{2} }} = 384.$$

This gave us a total of 1666 for the three study sites combined.

### Characteristics of participants

The study population comprised of children, both male and female up to 5 years and 4 months old attending the selected hospitals and presenting with clinical malaria as cases and apparently healthy pre-school and school children undergoing health screening as the controls. A total of 1666 patients were expected to be recruited as cases for the study per the sample size calculation but the final number recruited was 1629. A total of 445 children who fulfilled the above criteria were recruited as the controls. Children with clinical malaria presentation but had other medical condition in addition as well as those who were above 5 years 4 months were excluded from the study.

### Sample collection and laboratory analysis

Two millilitre of venous blood sample were taken from participants by venisection into an anticoagulant tube and transported in the appropriate medium from the various sample sites to the haematology laboratory of each hospital and analyses performed. Thin blood smears were done and stained with Leishman for morphological studies. Again, Thick and thin blood films were made and stained with Giemsa and examined for malaria parasites by two experienced independent microscopists as a way of ensuring proper quality control.

### Haematological analysis

For haematological analysis, whole blood specimen were analyzed for full blood count using ABX Pentra 60 C+ automated haematological analyzer and the result used to estimate the monocytes and lymphocytes ratio of each participant. Also the thin smears were examined to confirm the morphology of the cells.

### Parasitological analysis

Giemsa solution was prepared by dividing one part of the stock of Giemsa to ten parts of water. Both thick and thin films were prepared on clean, dry, grease free and labeled glass slide, with the thick film prepared closer to the frosted end and the slide left to air-dry. The whole slide minus the frosted end was dipped in the Giemsa solution for 10 min. The slide was then rinsed gently with water and placed vertically on a draining rack to air dry.

### Microscopic analysis

With the thick smear, a minimum of 100 fields were examined for the presence of blood parasites under oil immersion objectives. The parasites were counted against 500 leukocytes and parasites per microlitre of blood calculated against the total white cell count obtained from the full blood count using the formula: [number of parasites/500 WBCs × counted WBCs]. For the thin smears, a minimum of 200 fields under oil immersion objectives was examined for species identification.

### Monocytes, lymphocytes and neutrophil estimation

Results of the full blood count obtained from the automated haematological analyzer were used to estimate the monocytes, lymphocytes and neutrophil levels of each sample. The absolute count of the monocytes and lymphocytes level of each sample was entered into a calculator and the ratio was estimated by dividing the monocytes by the lymphocyte counts. The other ratios were calculated using the same formula.

### Data analysis

Data was collected using notebooks and transferred to a computer and kept confidential. They were later entered into Microsoft Word and analysed using Statistical Package for Social Sciences (SPSS, Version 20.0) and Graphpad prism. Normally distributed data were analysed using independent sample t-test expressed and as Mean ± SD. Skewed data were analysed using Mann–Whitney u test and expressed in Median and interquartile range. Spearman’s rank correlation was used to determine the correlation between occurrence of clinical malaria and the monocytes and lymphocytes ratio (M:L). Statistical significance was taken as p ≤ 0.05 with 95% confidence interval.

## Results

### Demographic description of study participants

A total of 1629 and 445 children aged 6 months to 5 years 4 months whose parents or guardians consented participated in the study as cases and controls, respectively. Out of the total number of cases, 35.3% were recruited from the Korle-bu Teaching hospital, 42.6% from the Sunyani Regional Hospital and 22.1% from the Wa Regional Hospital. Again out of the total number, 55% were males and 45% were females. Out of the total number of controls, 44.5% of them were recruited from the Korle-bu municipality, 35.5% from Sunyani municipality and 20% from Wa municipality. Here also, 57.8% were males and 42.2% were females (Table 1).

**Table 1:** Demographic description of study participants

Variables	Cases (n = 1629)		Control (n = 445)	
	Male	Female	Male	Female
Korle-Bu Teaching Hospital	316 (19.4%)	259 (15.9%)	115 (25.8%)	83 (18.7%)
Sunyani Regional Hospital	382 (23.4%)	312 (19.2%)	91 (20.5%)	67 (15.0%)
Wa Regional Hospital	198 (12.2%)	162 (9.9%)	51 (11.5%)	38 (8.5%)
Total	896 (55.0%)	733 (45.0%)	257 (57.8%)	188 (42.2%)

**Fig. 1:**
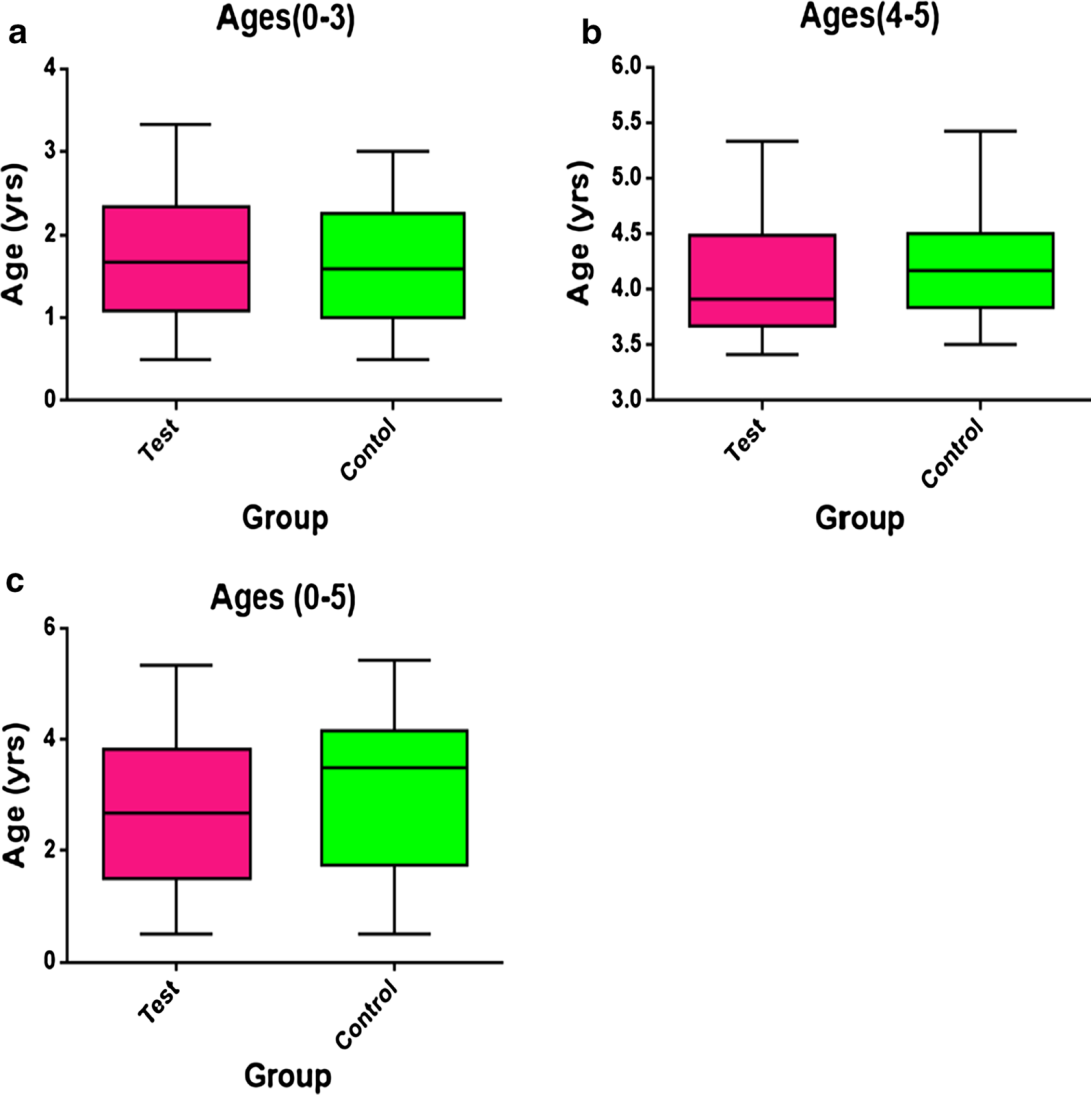
A figure of Box and Whiskers demonstrating the demographic description of study participants within age groups. Graph **a** compared the characteristic and ages of cases against controls in age category of 0–3 years. Graph **b** the category of 4–5 years and graph **c** all the ages combined

Out of the 1629 children recruited for the study as test group, 929 (57%) were within the age group of 0–3 years with an average age of 1.76 ± 0.32 and 700 (43%) were within the age group of 4–5 years with an average age of 4.13 ± 0.82. Also, out of the 445 children recruited as control group, 210 (47.2%) were within the age group of 0–3 years with an average age of 1.95 ± 0.75 and 235 (52.8%) were within the age group of 4–5 years with an average age of 3.81 ± 1.06 (summarized in Fig. 1). The average age of the total case group however stood at 2.14 ± 1.57 years and that of the control stood at 2.45 ± 1.62 years (Table 2).

**Table 2:** Demographic, clinical and laboratory variables of study participants

Variables	Cases (N = 1629)	Control (N = 445)	p-value
Demographic			
Average age (years)	2.14 ± 1.57	2.45 ± 1.62	
Clinical			
Temperature (mean ± SD) (°C)	37.9 ± 1.3	36.9 ± 0.5	
Pallor (%)	48	3	
Convulsing (%)	9	–	
Vomiting (%)	32	–	
Laboratory	Mean ± SD	Mean ± SD	
Haemoglobin (g/dL)	9.78 ± 2.4	11.95 ± 0.14	0.45
TWBC (× 10^9^/L)	7.77 ± 3.02	8.26 ± 3.40	0.58
	Median (IQR)	Median (IQR)	
Monocytes (× 10^9^/L)	0.60 (0.40–0.90)	0.40 (0.24–0.73)	0.0095*
Lymphocytes (× 10^9^/L)	1.91 (1.38–3.26)	3.42 (2.09–5.90)	< 0.0001 *
Neutrophils (× 10^9^/L)	4.53 (2.21–6.38)	3.41 (2.50–5.02)	0.2622
M:L ratio	0.31 (0.17–0.44)	0.11 (0.07–0.24)	< 0.0001*
N:L ratio	1.93 (0.82–3.63)	1.07 (0.59–1.87)	0.0011*
M:N ratio	0.14 (0.08–0.29)	0.12 (0.07–0.17)	0.113
Parasitaemia (/µL)			
Uncomplicated malaria	67, 317 (32,647–81,367)		
Severe malaria	112, 513 (109,801–143,246)		

*TWBC* total white blood cell, *M:L* monocytes to lymphocytes ratio, *N:L* neutrophil to lymphocytes ratio, *M:N* monocytes to neutrophil ratio

* Significant at p ≤ 0.05

Blood samples from the malaria patients showed positive blood smear for malaria parasites, while no parasites were found in the healthy control subjects. From the information obtained from Table 2, it indicates that there was a significant difference between lymphocytes of the case group and that of the controls with a p-value < 0.0001. There was also significant difference (p-value < 0.0001) between the monocytes to lymphocytes ratio of the cases and controls. Significant difference in neutrophil to lymphocyte ratios between the two study groups (p = 0.0011) was also observed. However no significance was established between the study groups as far as the Haemoglobin, total WBCs and monocyte to neutrophil ratios were concerned. In terms of parasitaemia, severe malaria and uncompleted malaria were categorized based on the parasite counts (Table 2).

**Fig. 2:**
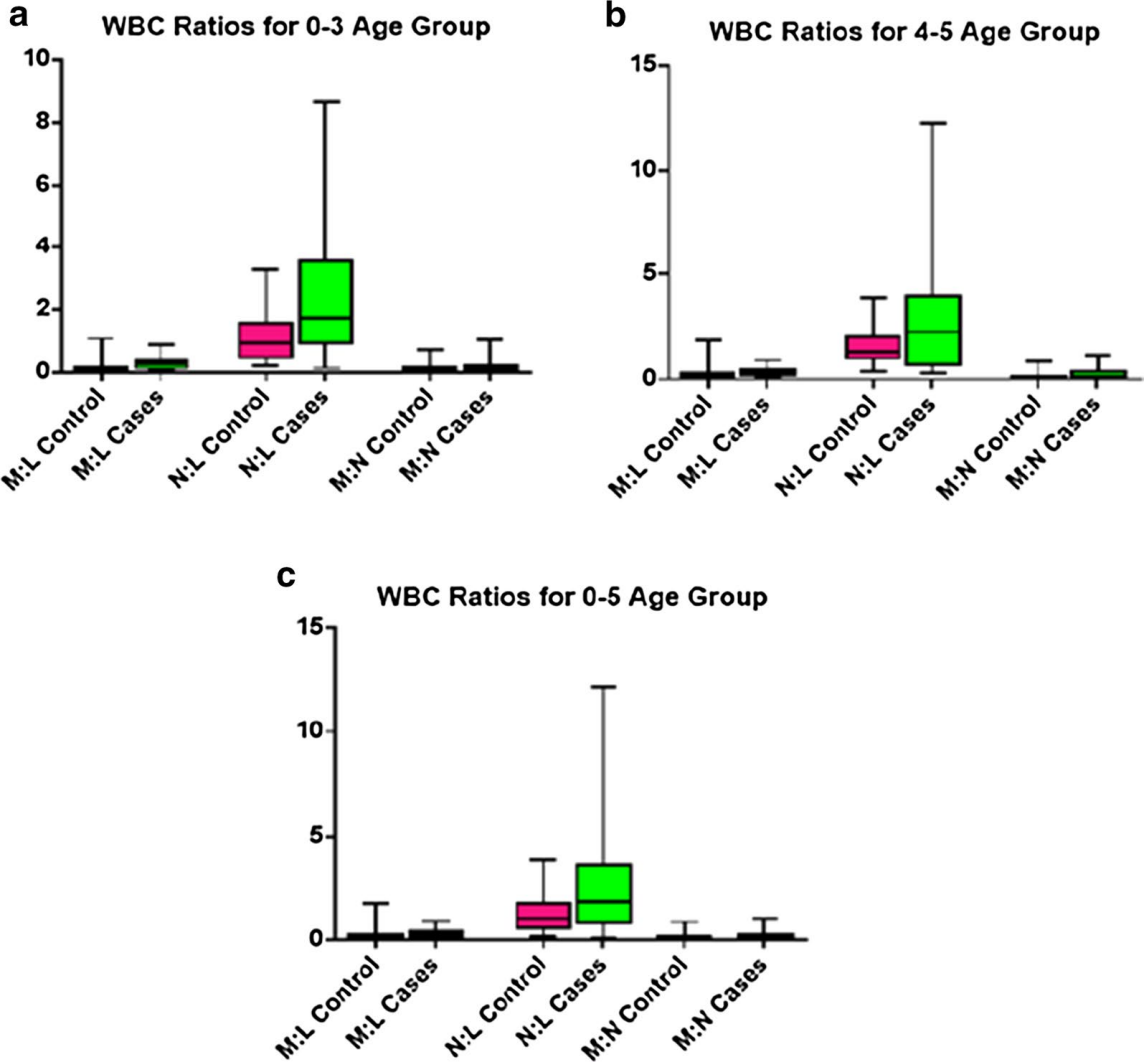
This is a figure of Box and whisker’s representing the different ratios obtained when the different white blood cells were compared

Comparison of haematological parameters according to age groups indicates that between both the ages of 0–3 and 4–5, there were significant associations between the lymphocytes (p = 0.0059 and p = 0.0345) and the monocytes to lymphocytes ratios (p = 0.0001 and p = 0.0008). Monocytes (p = 0.0175) and neutrophil to lymphocytes ratio (0.0087) showed significance among age 0–3 only whilst haemoglobin (p = 0.02) showed significant difference among age 4–5 years (Table 3). The different ratios obtained between the white blood cells were represented graphically with Box and Whiskers (Fig. 2).

**Table 3:** Haematological parameters of study participants according to age group

Age group	Haematological parameter	Cases	Control	p-value
		Mean ± SD	Mean ± SD	
0–3	Haemoglobin (g/dL)	9.55 ± 2.57	10.56 ± 1.45	0.43
	TWBC (× 10^9^/L)	8.93 ± 3.01	8.85 ± 3.17	0.81
		Median (IQR)	Median (IQR)	
	Monocytes (× 10^9^/L)	0.60 (0.40–1.00)	0.36 (0.21–0.84)	0.0175*
	Lymphocytes (× 10^9^/L)	2.72 (1.70–3.70)	3.70 (2.85–6.84)	0.0059*
	Neutrophils (× 10^9^/L)	5.50 (3.00–7.10)	3.34 (2.61–4.61)	0.0933
	M:L ratio	0.27 (0.13–0.39)	0.10 (0.06 –0.17)	0.0001*
	N:L ratio	1.73 (0.91–3.56)	1.05 (0.47–1.61)	0.0087*
	M:N ratio	0.15 (0.09–0.21)	0.11 (0.05–0.17)	0.1949
4–5		Mean ± SD	Mean ± SD	
	Haemoglobin (g/dL)	10.12 ± 2.06	11.30 ± 1.80	0.02*
	TWBC (× 10^9^/L)	7.74 ± 2.66	7.30 ± 3.66	0.66
		Median (IQR)	Median (IQR)	
	Monocytes (× 10^9^/L)	0.60 (0.30–0.82)	0.43 (0.28–0.71)	0.2254
	Lymphocytes (× 10^9^/L)	1.69 (1.30–2.39)	2.16 (1.47–4.95)	0.0345*
	Neutrophils (× 10^9^/L)	3.23 (1.87–5.60)	3.37 (2.24–5.20)	0.9773
	M:L ratio	0.38 (0.21–0.46)	0.14 (0.08–0.29)	0.0008*
	N:L ratio	2.24 (0.74–3.93)	1.26 (0.99–2.00)	0.0851
	M:N ratio	0.14 (0.07–0.37)	0.12 (0.07–0.17)	0.4145

* Significant at p ≤ 0.05

Again, from Table 4, haemoglobin and TWBC correlated positively with parasitaemia with values of r_s_ = 0.08 and r_s_ = 0.06 respectively although not significantly. On the other hand both monocytes and lymphocytes correlated negatively with values of r_s_ = − 0.01 and − 0.02 respectively but only the lymphocytes correlation was significant (p = 0.03).

**Table 4:** Summary of the haematological variables and their correlation to parasitaemia

Variables	Correlation co-efficient	p-value
Haemoglobin (g/dL)	0.08	0.45
TWBC (× 10^9^/L)	0.06	0.58
Monocytes (× 10^9^/L)	− 0.01	0.92
Lymphocytes (× 10^9^/L)	− 0.21	0.03*

* Significant at p ≤ 0.05

**Fig. 3:**
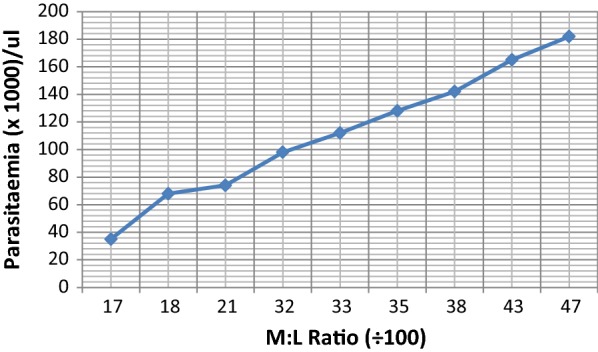
This figure is a plot of M:L ratio of the cases against the parasitaemia. It can be seen from the graph that as the parasitaemia was increasing so was the M:L ratio

Also as seen in Table 5, there was a significant positive correlation between the monocytes to lymphocytes ratio and the presence of parasites among the cases put together (p = 0.04). There was also a significant positive correlation between the monocytes to lymphocytes ratio and the level of parasitaemia within the age group of 0–3 years (p = 0.02) and 4–5 years (p = 0.03). The ratio was found to be increasing with increasing parasitaemia (Fig. 3).

**Table 5:** Correlation of monocyte to lymphocytes ratio and parasitaemia among the cases as well as the age groups

Variable	All ages	0–3 years	4–5 years
M:L ratio			
Correlation co-efficient	0.20	0.17	0.22
p-value	0.04*	0.02*	0.03*

* Significant at p-value ≤ 0.05

## Discussion

The study examined the monocyte to lymphocyte ratio during *P. falciparum* infections in children up to 5 years and 4 months. A total of 1629 children with an average age of 2.14 ± 1.57 participated in the study with parental/guardian consent. The number distribution from the study sites were as follows: Korle-bu Teaching hospital (35.3%), The Sunyani Regional Hospital (42.6%) and the Wa Regional Hospital (22.1%). Also, 55% of the participants were males and 45% were females. Four hundred and forty-five control subjects were also recruited for the study with an average age of 2.45 ± 1.62 years distributed as follows: Korle-bu Teaching Hospital (44.5%), Sunyani Regional Hospital (35.5%) and Wa Regional Hospital (20%) with 57.8% being males and 42.2% females (Table 1 and Fig. 1). Full or complete blood count was conducted on each participant and there was a significant decrease in the haemoglobin concentration in the *P. falciparum* infected children compared to those not infected with malaria (Table 2). The mean haemoglobin concentration was 9.78 ± 2.4, giving credence to the fact that malaria-induced immune responses contributes to accelerated lysis of both parasitized and non-parasitized red cells which in turn results in the increased anaemia witnessed in *P. falciparum* infected children [23, 24].

The monocyte and lymphocyte counts showed significant difference between the study groups (p-value = 0.0095 and 0.0001 respectively) even though there was no significant difference in the total white cell count between the groups (p-value = 0.58). Monocytes are essential component of the innate immune response as they act as a link to the adaptive immune system through the presentation of antigen to lymphocytes. Subsequently, during an infection, there is variation or derangement in the monocytes and lymphocytes numbers which also affect their functions [25]. The study found that the monocytes to lymphocytes ratio associated with all ages showed significant correlation with parasitaemia with a p-value of 0.04. Further, when the ratio was correlated with the age groups, significant correlations were established as well, with a p-value of 0.02 and 0.03 respectively (Table 5). It is well known that young children and pregnant women are more vulnerable to disease and death by malaria and the results obtained from this work indicated that. In fact, the increased M:L ratio is an indication of reacting immune system and as it correlates with parasitaemia, it also point to the fact that the ratio can be used as a prediction of parasitaemia. It was also found that the higher the M:L ratio, the higher the parasitaemia which can result in severe malaria and subsequently death (Fig. 3). Usually, after age 5, an individual would have reached a protective semi-immune status due to the fact that following repeated attacks of malaria, a person develops a partial protective immunity to the disease. Even though there is no clear concept as to how this protection works [26].

Furthermore, the relative frequency of monocytes to lymphocytes in blood circulation reflects one’s capacity to initiate an effective immune response against an ongoing infection. It can therefore be postulated that the monocytes to lymphocytes ratio can be a marker of an individual’s capacity to mount an effective immune response against malaria infection [15]. Boström et al. [27] suggested that a balance of pro and anti-inflammatory immune responses following exposure to malaria parasites may be an important factor in determining clinical protection. This is because the innate immune responses are initiated through pro-inflammatory cytokines and this is thought to contribute to the initial control of parasitaemia following infection by *P. falciparum.* This antiparasitic immune response though requires tempering by the adaptive immune response if effective immunity to clinical malaria is to be achieved [15].

Warimwe et al. [15] have shown that a high ratio of monocytes to lymphocytes (M:L ratio) in peripheral blood at a cross-sectional survey correlates with increased susceptibility to clinical malaria in older children (median age 4.5 years) during follow-up. This correlation between M:L ratio and clinical malaria risk was evident even after accounting for inter-individual differences in the levels of antibody correlates of clinical immunity in the study population. Some other researchers have also reported that the ratio of monocytes to lymphocytes in peripheral blood largely correlates with the extent of disease in both humans and some primates. Although the numbers studied were small and the strength of the conclusions that could be reached in humans were conceded to be modest, the works have been expanded further to prove that can be the case [28, 29]. A limitation in the current study worthy of mention was that the monocyte to lymphocyte ratio and parasite positive or negative status was measured at a single time point which is likely to introduce bias in the study due to variation in the monocytes and lymphocytes level in an individual at a specific time. Also the use of only microscopy based parasite detection method, might have missed some low parasitaemia infections in children and hence classified as negative. It would also have been interesting if it was possible to measure cell counts at the beginning, during and after the disease.

## Conclusion

A high monocyte to lymphocyte ratio might indicate a predominantly pro-inflammatory immune response that renders an individual susceptible to clinical malaria and increased parasitaemia. The main conclusion as far as this work is concern is that increased monocyte to lymphocyte ratio correlates positively with the presence and increased parasitaemia among children up to 5 years of age. As parasitaemia increases, development of severe malaria is almost always the consequence. The outcome of this work therefore indicates that when M:L ratio is used with other clinical and haematological parameters, it can significantly improve malaria diagnosis and further assist with treatment choices.

## Abbreviations

HIV: human immunodeficiency virus
KBTH: Korle-bu teaching hospital
SD: standard deviation
SRH: Sunyani Regional Hospital
SPSS: Statistical Package for the Social Sciences
TWBC: total white blood cell count
WRH: Wa Regional Hospital
WHO: World Health Organization
